# A blended neurostimulation protocol to delineate cortico-muscular and spino-muscular dynamics following neuroplastic adaptation

**DOI:** 10.3389/fneur.2023.1114860

**Published:** 2023-06-15

**Authors:** Filip Stefanovic, Julian A. Martinez, Ghazala T. Saleem, Sue Ann Sisto, Michael T. Miller, Yaa A. Achampong, Albert H. Titus

**Affiliations:** ^1^Department of Biomedical Engineering, State University of New York at Buffalo, Buffalo, NY, United States; ^2^Department of Rehabilitation Science, State University of New York at Buffalo, Buffalo, NY, United States; ^3^UB Department of Biomedical Engineering, State University of New York at Buffalo, Buffalo, NY, United States

**Keywords:** TMS, tDCS, NMES, corticospinal, motor control

## Abstract

In this paper we propose a novel neurostimulation protocol that provides an intervention-based assessment to distinguish the contributions of different motor control networks in the cortico-spinal system. Specifically, we use a combination of non-invasive brain stimulation and neuromuscular stimulation to probe neuromuscular system behavior with targeted impulse-response system identification. In this protocol, we use an in-house developed human-machine interface (HMI) for an isotonic wrist movement task, where the user controls a cursor on-screen. During the task, we generate unique motor evoked potentials based on triggered cortical or spinal level perturbations. Externally applied brain-level perturbations are triggered through TMS to cause wrist flexion/extension during the volitional task. The resultant contraction output and related reflex responses are measured by the HMI. These movements also include neuromodulation in the excitability of the brain-muscle pathway via transcranial direct current stimulation. Colloquially, spinal-level perturbations are triggered through skin-surface neuromuscular stimulation of the wrist muscles. The resultant brain-muscle and spinal-muscle pathways perturbed by the TMS and NMES, respectively, demonstrate temporal and spatial differences as manifested through the human-machine interface. This then provides a template to measure the specific neural outcomes of the movement tasks, and in decoding differences in the contribution of cortical- (long-latency) and spinal-level (short-latency) motor control. This protocol is part of the development of a diagnostic tool that can be used to better understand how interaction between cortical and spinal motor centers changes with learning, or injury such as that experienced following stroke.

## Introduction

Neuromodulation of corticospinal excitability has recently been shown to be an effective tool to increase the efficacy of rehabilitation outcomes (1). For motor control applications, the effects are most often explored as a collective system to identify causal relationships between the brain and muscle, creating a black box-type understanding. In other words, we can relate the inputs (i.e., issued control commands) and outputs of the system (e.g., motor evoked potentials, biomechanics), but the basis of neurophysiological function remain incompletely classified due to the complexities of the corticospinal system. As such, here we present a novel blended neurostimulation protocol that aims to delineate cortical and spinal level processing in specific motor control tasks during volitional wrist motion.

Non-invasive brain stimulation (NIBS) or neuromodulation are ever increasing tools used to improve neuroplastic outcomes in motor neurorehabilitation. Low-cost, safe options such as transcranial direct current stimulation (tDCS) are especially popular due to their ease of use, and economical ubiquity. Generally, tDCS is widely acknowledged as having long-term modulatory aftereffects on cortical excitability that are dependent on dosing (2). For example, repetitive use of tDCS has facilitated improvement in neuroplastic motor relearning (3–8) in multidimensional movement parameters such as peak and accuracy of movement. Generally, applications of tDCS are used under varying assumed mechanisms: (a) depolarize cortical neurons (anodal tDCS) in order to increase cortical excitability; (b) hyperpolarize cortical neurons (cathodal tDCS) to decrease cortical excitability; or (c) as a sham neurostimulation, where the applied voltage/current is low enough to prevent a neural response (9–13). However, the success of tDCS applications is highly variable likely due to inter-individual neuroanatomical differences, the montage, the dosage, as well as unknowns due to gaps in the scientific and functional knowledge related to its application (14–21). Similarly, recent research has shown that tDCS mechanisms can change in effect depending on these variables. For example, the neuroanatomical structures of neurons can cause hyperpolarization at the anode, and depolarization at the cathode (22–24). Ultimately, this can lead to variance in the intended behavior of the applied mechanisms. Largely it is agreed that if the variability can be minimized, significant improvements can be made to tDCS usage and success rate—as such, more robust protocols can be part of the solution to this problem. Success may lead to individualization of tDCS based on baseline inputs by the user.

Another form of NIBS that performs similarly to tDCS is transcranial magnetic stimulation (TMS) (25). TMS is generally considered to have better spatial and temporal resolution (26) in its application. It can be combined with EEG and/or fMRI mappings to optimize stimulation localization. However, tDCS and TMS mechanisms of action are considerably different (27). For example, for TMS, a coil is used to focus a field to induce action potentials compared to tDCS which uses surface electrode pairs to inject an electric field that impacts neuronal activity. Also, TMS is shown to reach deeper neural tissues than those typically affected by tDCS (28). While both can neuromodulate, only TMS can elicit action potentials (29–32). Thus, TMS provides an opportunity for brain level triggering of the motor evoked potential (MEP), while concomitant tDCS provides a tool to precondition a neuromodulated response (33).

These NIBS based modalities are driven primarily at the cortical level to trigger feedforward mechanisms that drive the brain-motor response. However, feedback plays a significant role in not only tuning motor control responses, but also in adjusting motor responses due to dynamic changes, as well as in motor learning (34–36). For example, spinal-level motor control centers are locally responsible for modulating short-latency feedback found at the spinal level. A common way to trigger these spinal-level responses are through the use of neuromuscular electrical stimulation (NMES) (37). In these instances, NMES can activate sensory and/or motor neurons that trigger contraction in a muscle fiber (38, 39). Phasic NMES stimulation shows modulatory effects on spinal-excitability, implying that spinal-driven responses can be modified based on dosing at the local level (i.e., time, frequency). But more significantly, this sensory-motor learning initiated at the muscle-spinal-level can also modulate sensorimotor activity at the cortical level (40, 41). These observations suggest that cortical-level neuromodulation affects downstream spinal responses, while spinal-level neuromodulation affects cortical-level motor learning. There is also a subset of NMES that is called functional electrical stimulation (FES) that operates using a similar concept (i.e., stimulation of a muscle and triggering spinal-level motor control pathways). However, FES is a form of NMES that is applied during a functional task and aids in specific neurorehabilitation for that task-based motion.

A question remains, however, as to how these neurostimulation approaches work together. Recent studies have shown that a combination of NIBS and NMES have beneficial effects in post-stroke and other neurorehabilitation. Specifically, Satow et al. (42) demonstrated that a combined tDCS and NMES protocol improved the outcome in post-stroke gait rehabilitation. This appears to suggest that the neuromodulation provided through tDCS can have a response effect on the spinal-level control, or at least facilitate related motor relearning at the cortical level. These findings were observed by several others (43–45). Schabrun et al. (46) explored the possibility of measuring if these effects had a linear (summative) effect on M1 enhanced excitability but found that the behavior was much more complex. Regardless, Shaheiwola et al. (47) found similar improvements during clinical trials, noting that tDCS enhanced FES when explored through a randomized test. Interestingly, the study subjects had their MEPs measured through TMS at the start and end of the protocol, indicating that those who underwent anodal tDCS during FES showed significant difference from those who underwent FES with a sham tDCS. When exploring individual neurostimulation locations, it was found that cortical level neurostimulation could outperform spinal level applications (48). Similar positive outcomes were measured in TMS effects on NMES (49–51), but in all cases further exploration was suggested as the corticospinal mechanisms behind the outcomes were not fully understood.

In our paper, we propose a novel blended neurostimulation protocol that combines tDCS, TMS, and NMES for the purpose of probing the corticospinal network, and delineating cortico- and spinal-level motor contributions. Here, we will describe our protocol that uses: (a) tDCS to neuromodulate cortical level motor formation and affect motor task urgency and motor response time (4); (b) TMS to elicit a brain-motor perturbation affecting the feedforward motor controller; and (c) NMES to trigger spino-motor perturbations and reflexive motor responses. This multidimensional neurostimulation strategy is part of a larger study that aims to separate corticospinal motor control into functional cortico- and spinal-level blocks, in an effort to build a more patient-specific computational model for clinical applications.

## Materials

### Development of the human-machine interface

**Figure 1:**
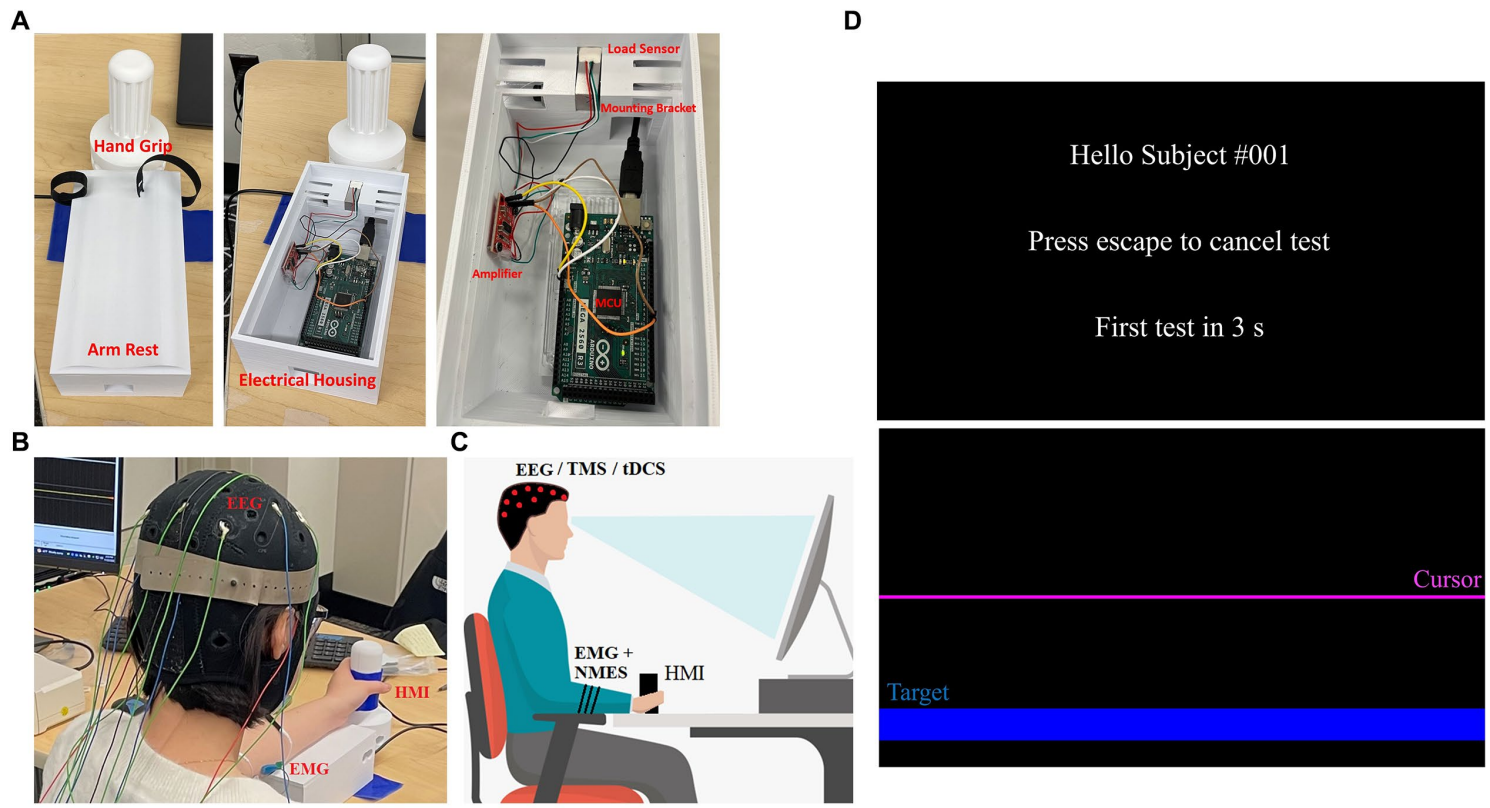
HMI and protocol set up. **(A)** Hand grip and arm rest are shown in its operational state (left) as well as with the arm rest remove to show the electronics (middle). The electronics—Arduino, amplifier, sensor—and how they are positioned are shown (right). **(B)** An individual seated using the HMI while wearing an EEG cap, EMG and NMES electrodes. **(C)** A depiction of the system setup including posture required for the protocol—red dots indicate EEG/TMS/tDCS electrode placements, while black stripes on arm indicate EMG and NMES electrode placement. **(D)** GUI for the HMI is shown with the welcome screen (top) and task screen (bottom). The task screen shows the target location (blue) as well as the cursor (purple) that moves when the wrist is flexed/extended.

The human-machine interface (HMI) was developed in-house using 3D printing, various open-source electronics, and a freely available graphics user interface (GUI) builder for MATLAB. The HMI was designed using CAD software (Fusion 360) and included a handle, armrest, and housing unit (see Figure 1A). Dimensions of the system are: 200 mm × 70 mm × 110 mm (arm rest and housing for electronics), and 143 mm × 25 mm diameter (hand grip). The drafted design was 3D printed using PLA filament with a Robo R2 printer. The handle is connected to the housing unit using a 10 kg Straight Bar Load Cell (TAL220), so that when a user attempts to move the handle a force is measured. The Load Cell data are amplified using a SparkFun Load Cell Amplifier (HX711) and are then sent to an Arduino Mega 2560 R3 (MCU) for data collection and processing. The handle was designed in a such a way so that the wrist rests above the load cell, and the torques produced in the joint correspond to the loading in the sensor.

**Figure 2:**
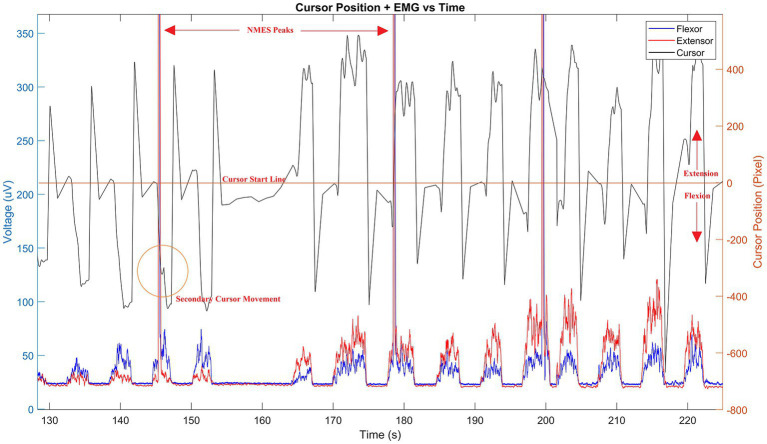
Sample data stream of HMI. Cursor pixel location (black line) is shown during use with corresponding wrist muscle flexion and extension EMGs (blue and red lines, respectively) superimposed. Resulting NMES peaks are shown in measured EMG and a corresponding cursor shift (orange circled area) visible by delayed secondary peak response following stimulation. The time offset between stimulation and cursor movement define the computational properties of the corticospinal controller as described by Equations 1–9. Time is given in seconds—only a portion of the signal is shown to emphasize the sample results.

The Arduino MCU is connected to a PC running an open-source MATLAB module known as Psychtoolbox (52, 53). We built a custom GUI (Figure 1D) for this protocol that instructs the user when to relax and when to perform tasks with their non-dominant hand. For example, when the GUI starts, the user is prompted that the test will be in 5 s (relax phase). When the relax phase completes, a target is presented on screen that the user must reach by exerting a force on the HMI hand grip, which is detected by the loadcell. This force is generated by the gripping hand due to the contracting wrist muscles. Thus, these forces generated by the hand via flexion and extension move the cursor in a downward or upward direction, respectively. The cursor sits at the center of the screen when the user applies no force to the HMI and will move away from this zero-point as the applied force increases in either direction. To complete the movement task, the user must keep the cursor on target for 3 s, after which the target is removed from screen and the study participant is asked to relax again for 5 s. When relaxed, the screen cursor goes back to the center of the screen. This procedure is repeated 60 times until completion, the first 20 being wrist flexion movements for random targets on the bottom half of the screen, followed by 20 wrist extension tasks for targets on the top half of the screen and lastly 20 alternating targets. An additional 10 s rest is given after each completed section. The task is performed naturally as per the user’s ability, and periodically under TMS perturbation or NMES perturbation. Specifically, ~25% of the movements are done under neurostimulation in order to perturb the neuromuscular system in random intervals between 2 and 8 repetitions. Sound cues at the beginning of each test create EEG spikes that reflect the beginning of each test. To account for differences between users, an optional calibration step tasks each user with reaching a separate set of targets at higher difficulty than the main test. During this step, data are taken corresponding to HMI control in each direction individually, and are used to determine difficulty and sensitivity to be used in the main program for consistent control from user to user. Calibration can be skipped, and a preset sensitivity can be used, or sensitivity can be manually altered to make the tasks more or less difficult as needed. Time and cursor data are saved after each individual test, and the entire data stream is saved once a whole test session is completed (Figure 2).

During experimentation, the HMI is used with Trigno Avanti EMG sensors (Delsys Inc.) (see Figures 1B,C). A standard 10/20 EEG electrode layout is also used to measure cortical activity during motions. Additionally, a standard posture for task performance is used. The participants are seated upright, and the forearm is in mid-prone position with neutral wrist and fingers flexed when the handle is gripped (Figures 1B,C). The participant is instructed to hold the elbow and shoulder angles as close to 90° as well as to remain in that posture throughout the trial. Participants are instructed to also grip the device firmly in a neutral grip. In the event of a posture change, they are promptly asked to bring their posture back to the initial position. Their arm is strapped into the HMI, so it remains centered and flat across the top of the device, and a cloth is placed over the arm/hand so that they cannot visually observe their movements.

Noise can potentially interfere with small magnitude biopotentials, such as EEG, captured in this protocol. To address these, standard filtering methods can be applied to remove known noise sources (60 Hz line noise, motion artifacts, etc.). Additionally, no startling effects are expected due to our ramped up stimulus, so this is not expected to create additional noise.

## Methods

### NMES protocol

To assess spinal motor control network excitability, we will apply NMES on the extensor carpi radialis (ECR) and/or flexor carpi radialis (FCR) during the HMI movement task. The applied NMES will trigger H-reflex in the muscle and resultant MEP will be captured using the Trigno Avanti EMG sensors with two sensors placed on the ECR and two on the FCR muscles. The location of the ECR is found by using a motor map derive from a cursor pixel/EMG relationship as shown in Figure 2. Similarly, the muscle belly is identified by having the subject place their forearm down on a flat surface with their palm down, then extending their wrist towards the thumb and back. The FCR is found by using the motor map, and/or having the subject place their forearm on a flat surface with their palm up to identify the muscle belly. To do so, the subject brings their thumb to their middle finger and flexes their wrist so their fingers point to their elbow. While the subject is in the flexed position the contracted muscle belly is found by touching the muscle. The NMES will be applied over two channels (one for ECR and one for FCR) and will be produced using a Hasomed RehaStim in Sciencemode controlled via Labview (settings—Baudrate: 115200; Data Bits: 8; Parity: None; Stop Bits: 2; Flow Control: CTS). Labview triggers the stimulation when the cursor begins entering the target area. Stimulation levels are determined using a ramp up, initially, with a 5 mA amplitude 250 μs pulse, and then incremented by 5 mA until reaching the individuals maximum comfort range. For repeated NMES, we use an amplitude that is 85% of that maximum (although some may require higher for a more profound effect). To verify the NMES triggered MEP, the stimulation should evoke a contraction that is at least 25–30% of a maximum voluntary contraction (54) as measured using the HMI which minimizes the likelihood of a startling effect.

### TMS protocol

TMS (MAG & More) will be used to probe the neuromuscular controller at the cortical level using a series of motor evoked potentials (MEP). The MEP will perturb the neuromuscular movement using the HMI to affect a feed-forward external modification to brain-level issued motor commands, and to act as a disturbance in motor learning.

The anatomical landmarks for TMS localization will be identified using 3-Tesla functional magnetic resonance imaging (fMRI) of the wrist primary motor cortex area (M1). To do so, all subjects will undergo wrist activation experiments during fMRI consisting of three 30-s periods of rest alternating with three 30-s periods of wrist extension and flexion at a rate of approximately 0.5 Hz (55). Brainsight neuro-navigation will be then be used with the generated fMRI mappings to localize the wrist ECR and FCR target hotspots. High field strength at the localized wrist extensor and flexor hotspots will be verified by measuring 10 consecutive TMS-evoked peak-to-peak MEPs with an average amplitude of 0.5-1 mV at a rate of approximately 0.5 Hz (56). More than 10 consecutive MEPs can be used, but here, we implement 10 since this amount provides a high reliability (57, 58). Although TMS exhibits some variability, the MEP amplitude inconsistencies that are expected over time do not affect this protocol’s ability to assess corticospinal behavior.

Finally, it is worthwhile to note that TMS and NMES are not required to be used simultaneously, as the motor response can be measured in succession as described below. They can be used either in subsequent movements or following a > 60 ms voluntary stimulus evoked delay window (59).

### tDCS protocol

When considering the HMI movement task, we will also apply anodal tDCS to modulate motor control excitability to determine how movements are scaled (60). To perform the task under tDCS we apply similar methods to those presented in earlier studies, such as by Lackmy-Vallee et al. (61). Specifically, we will use a 10/20 EEG guided placement verified by our TMS to determine anode placement. We will apply a 2 mA current which corresponds to the mean intensity threshold ascertained in a study that examined the functional architecture of the motor homunculus for tDCS (62), and similar to the intensity used in other studies (63). Anodal stimulation will be applied for 15 min prior to performing the movement task with the HMI. The anode will be placed over M1 (targeting wrist extensor or flexor) while the cathode is placed over the contralateral supraorbital area. Here, we apply tDCS prior to the motion but it has been shown to be effective when administered before the task as well. Thus, precise tDCS stimulus/perturbation timing during the task is not required.

We also apply sham stimulation for control purposes by placing electrodes on the same positions and stimulating for 120 s at the start and 30 s at the end. The sham stimulation is applied with a 2 mA current based on previously accepted methods (61–63).

The tDCS will be introduced into the protocol after TMS as the mechanism as it (anodal tDCS) entails depolarizing the neurons to increase the probability of action potential—TMS is used first to induce an action potential. Research has also shown that certain neurons that are inactive respond strongly to the TMS. tDCS will be used in an online fashion where anodal stimulation will be provided during the task.

Electrode sizes of 5 cm × 7 cm are used.

### The blended NIBS-NMES method

Our blended neurostimulation protocol combines the aforementioned tools into a single combined protocol aimed at isolating cortico-spinal neuromuscular control pathways and to measure the distinct pathway features related to neurorehabilitation intervention.

**Figure 3:**
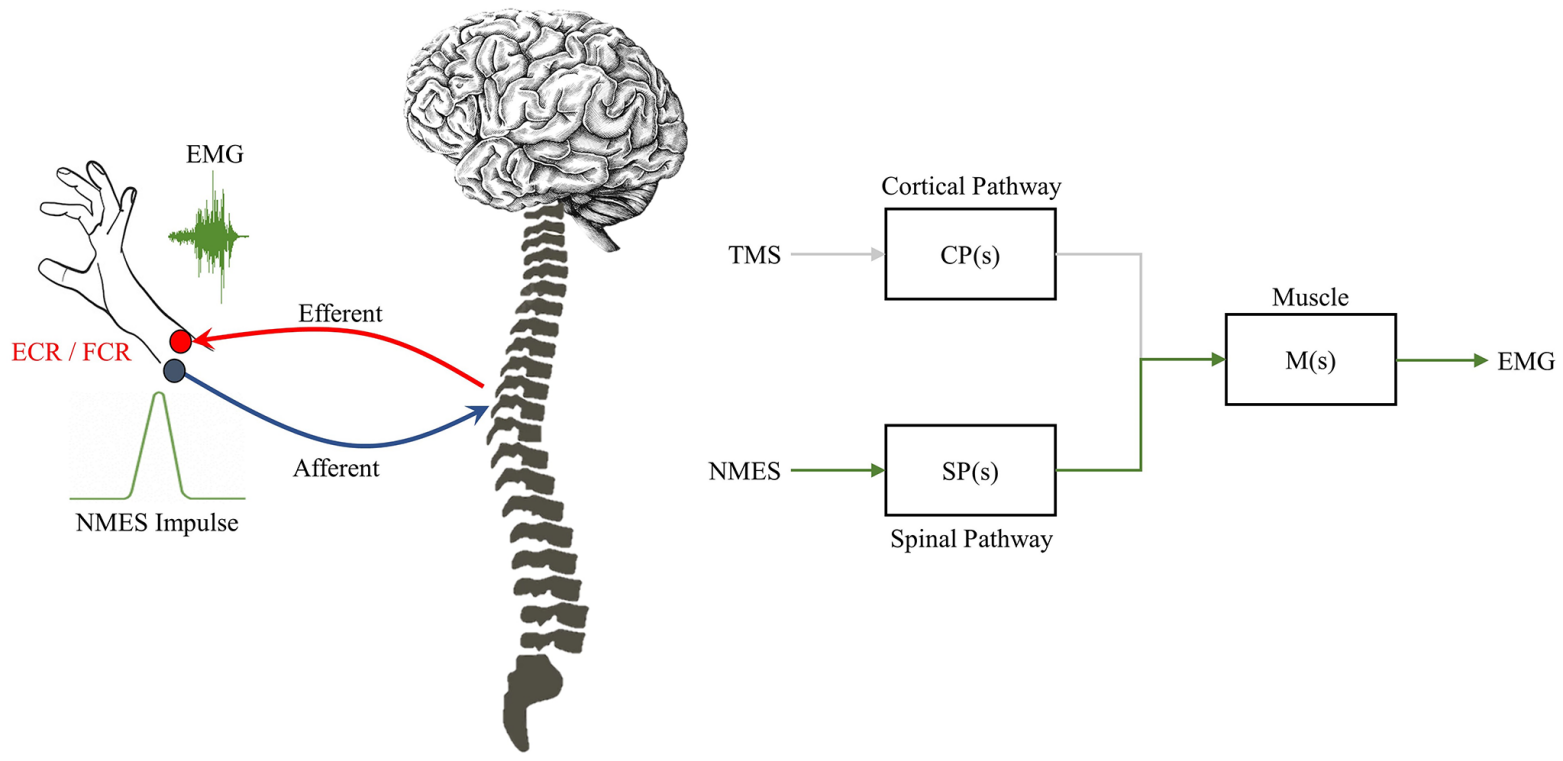
NMES impulse pathway. NMES typically first triggers the sensory-afferent pathway, then affects the homonymous efferent pathway to generate a MEP. The spinal-muscle section in this mode is shared with the feedforward TMS triggered pathway, but does not share the descending brain-spinal cord network elicited through TMS.

During a volitional motor task, the application of TMS generates a feedforward perturbation onto the motor control dynamics. Specifically, the resultant MEP change is thus measured by a change in contraction amplitude, a change in the phasic activity in burst contractions, as well as in the co-ordination effect on a multi-muscle system (ECR vs. FCR). The effect of the generated TMS perturbed and volitional MEPs is modulated by tDCS such that motor excitability increases to affect the feedforward mechanisms of the motor controller. In this way our combined TMS + tDCS protocol provides an impulse-response probing tool to identify unique person-specific feedforward motor behavior (64). Where, impulse-response dynamics are ubiquitous for their use in understanding wide-band frequency behaviors of complex time-invariant systems. Thus, the TMS generated impulse creates a neurological mapping of kinematic-EMG dependencies based on the cortically generated motor control plan. Whilst, tDCS neuromodulation of the M1 provides system modification (through motor excitability) that would be captured in a subsequent impulse-response measurement. As a result, this gives us a tool to identify the causal relationships between brain-muscle pathways, and more importantly, how they change over time during motor learning (65, 66) (see Figure 3). Data acquisition and stimulation are controlled with a single computer, time-synced system, and thus all data are time stamped to ensure synchronization.

Additionally, the NMES impulse at the spinal-level creates a secondary measurable dynamic response. NMES applied at the muscular level triggers direct motor response (M wave) and/or a closed-loop afferent-spino-muscular response (NMES ➔ muscle) that captures the neural dynamics related to the motion (also see Appendix) (38). For example, M1 issued motor commands are a feed-forward representation of the movement strategy, thus by perturbing only the spinal-level motor controller the cortical feed-forward mechanisms remain intact, but spinal-level computations are altered. It is important to note that although the afferent-to-efferent pathway shown in Figure 3 includes the sensory-motor feedback loop in the motor controller, the NMES triggers short latency neuromuscular stimulation in a feedforward matter (i.e., direct path from NMES to muscle stimulation) and thus does not represent a closed loop mechanism. The true feedback response comes after the initial NMES stimulation (M wave), following spinal or cortical level processing (H-reflex or F wave). Additional information on the behavior of these mechanisms can also be found in our earlier computational studies of the corticospinal system (4, 67–69). The NMES thus provides impulse-response dynamics of short-latency sensory-modified motor control strategies irrespective of cortical driven movement formation (70).

In terms of the system response, the dynamic control motor outputs, MO, of these pathways are defined using standard representative systems:

(1) $$MO_{TMS}=M\left(s\right)CP\left(s\right)R_{TMS}\left(s\right)$$

(2) $$MO_{NMES}=M\left(s\right)SP\left(s\right)R_{NMES}\left(s\right)$$

**Figure 4:**
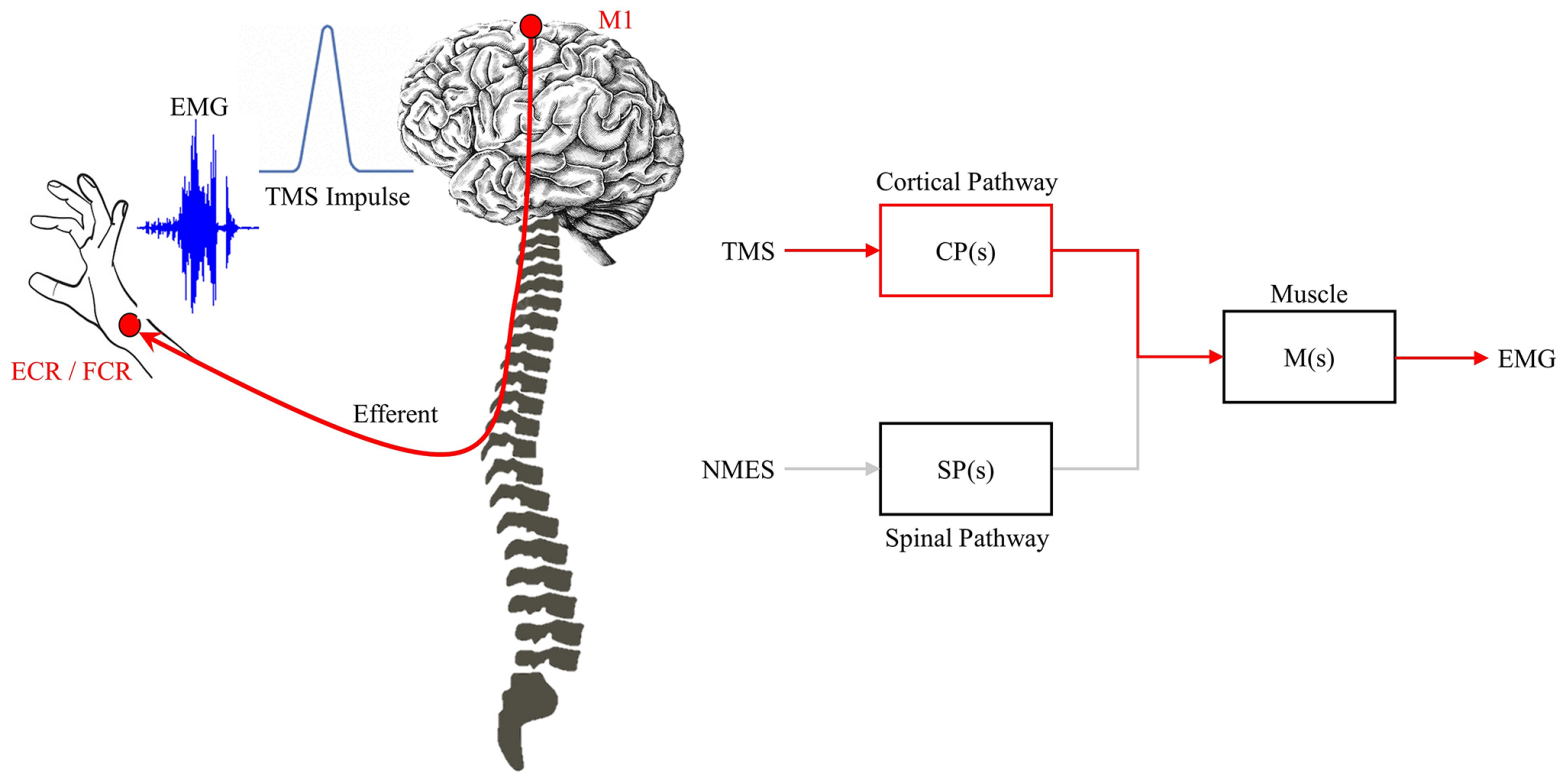
TMS impulse pathway. TMS triggers the feedforward network to generate a MEP. The impulse-response characteristic relates TMS-EEG and is driven primarily by the feedforward/efferent pathway (red).

Where, R is the input function. Individually these system representations denote their unique stimulation-response pathways, e.g., cortico-muscular or spinal-muscular, CP is the cortical pathway, SP is the spinal pathway, as shown in Figures 3, 4. If both pathways are known through application of TMS and NMES, their relative contributions can be discovered mathematically such that:

(3) $$\begin{array}{c} MO_{TMS}-MO_{NMES}=\Delta_{MC}=M\left(s\right)CP\left(s\right)R_{TMS}\left(s\right)\\ -M\left(s\right)SP\left(s\right)R_{NMES}\left(s\right) \end{array}$$

Such that the change in motor command dynamics, ∆*_MC_*, is defined by:

(4) $$\Delta_{MC}=M\left(s\right)\left[CP\left(s\right)R_{TMS}\left(s\right)-SP\left(s\right)R_{NMES}\left(s\right)\right]$$

Or, given that the input is a normalized function:

(5) ΔMCR(s)=M(s)[CP(s)−SP(s)]

In other words, the measured difference in the change in motor response is proportional to the difference in motor control input from cortical, CP(s), and spinal-level, SP(s), commands. This represents an important concept to delineate hierarchical control paradigms. Specifically, the blended neurostimulation protocol enables the shared neuromuscular controller to be probed in such a way to identify unique cortical- and spinal-level contributions to the motor control strategy.

Similarly, if $\Delta_{MC}/R\left(s\right)\;$ measures the motor level activity, we can infer unique CP or SP contributions to the motor control outcome as well. For example, by taking Equations 1–5:

(6) MOTMSR(s)−ΔMCR(s)=M(s)CP(s)−M(s)[CP(s)−SP(s)]

(7) $$\frac{MO_{TMS}-\Delta_{MC}}{M\left(s\right)R\left(s\right)}=SP\left(s\right)$$

And, by taking Equations 2 and 5:

(8) MONMESR(s)+ΔMCR(s)=M(s)SP(s)+M(s)[CP(s)−SP(s)]

(9) $$\frac{MO_{NMES}+\Delta_{MC}}{M\left(s\right)R\left(s\right)}=CP\left(s\right)$$

Thus Equation 7 shows that the spinal-level isolated impulse-response, SP(s), can be measured by comparing the response characteristics due to TMS and NMES. Similarly, Equation 9 shows that the cortical-level isolated impulse-response, CP(s), can also be measured in a similar way. However, what is important to identify as part of these equations that the muscle pathway motoneuron effect, M(s), is removed. This reduces controller block sizes, and separates the cortical and spinal functionality before motor execution. In other words, the unshared portions of the neural impulse-responses can be investigated [CP(s): brain to spinal cord pathway; and SP(s): afferent sensory pathway]. This is novel in that the complexity of delineating cortical and spinal systems is exceptionally difficult due to their shared endpoint networks. Based on our earlier studies, these computational representations of the complex non-linear motor controller have been validated with similar NIBS based studies measuring motor response times (4, 67–69). Specifically, although a linear approach is taken above, the components of the system are highly non-linear and have shown a robust ability to emulate the corticospinal motor controller.

### Expected outcomes

The development of this presented neurostimulation method is part of a larger study that aims to create the first corticospinal model of the neuromuscular controller. As a result, using our blended protocol we will achieve two representations of the motor controller, its issued commands, and their dynamic responses using TMS (along with cortically modulated TMS via tDCS) and NMES. These measured behaviors will thus give us impulse-response behaviors of long latency feed-forward neuromuscular systems (cortico-muscular pathway) as well as short-latency feedback control modifications (spino-muscular pathway) in the motor task. Used jointly, the blended method provides a tool to probe the neuromuscular system and to determine brain- and spinal-level contributions to motor control. However, what is more, is that these impulse-response dynamics will be person-specific, and response dynamics will change over time with motor learning. For example, the M1 issued feedforward control will change over time as an individual learns a task or through tDCS neuromodulation (71), and thus the impulse-response generated via TMS will change to reflect that (72). Similarly, spinal-level NMES impulse-dynamics measure changes in spinal network topologies during motor learning independently of cortical level learning (73).

In the short term we will apply this blended method on healthy subjects to explore variation in cortical- vs. spinal-level relative contributions, and how it changes longitudinally with time. What we expect to find is that as an individual becomes more adept at a motion task, the interconnection of corticospinal pathways will change; e.g., increased corticospinal functional connectivity (74). So, with motor learning the feedforward M1-Muscle pathway will be more pronounced in learning motion behaviors, as sensory dependencies decrease in motor command formulation under these conditions (75).

Later, we will apply this new method as part of a clinical-based study to explore motor re-learning for neurorehabilitation. Patients participating in the study will undergo this protocol in at least three milestones of their neurorehabilitation protocol: (a) Immediately following stroke, at the start of the treatment; (b) at an approximate half-way point during their rehabilitation plan; and (c) at the end of the neurorehabilitation plan (either in a clinical or at-home setting). We will use the blended neurostimulation protocol to measure changes to the neuromuscular controller from baseline, and track their progress over time. By measuring impulse-response dynamics of the corticospinal system, we will identify how these control pathways change—specifically, how they issue motor commands during re-learning—over time. We expect, much like with healthy subjects, there will be an increase in the feedforward pathway such that CP(s)—SP(s) (see Equation 5) becomes more positive. We also expect to see that the peak MEP variability associated with TMS impulse decrease over time as reported in earlier studies. Inter-test repeatability is inconsistent across NIBS studies, but it has been shown that the variability decreases with repeated stimulation and/or neuromodulation (not necessarily the peak amplitude of the MEP) (76). This coincides with what we expect in motor learning, since as the feedforward controller improves, less internal variation will occur in the issued motor command and subsequently less emphasis given to sensory-driven command formulation.

Ultimately, this new method gives clinicians and scientists a unique template to understand variable across populations, changes in individuals over time based on motor learning, and allows healthy and diseased states to be classified cross-sectionally.

### Study limitations

Some of our suppositions of the measured impulse responses of the system are simplified representations such as in Equations 1–9. In reality, the corticospinal system is a highly non-linear network that cannot be represented by simpler linear time-invariant representations. However, although the computational approach is linear, the components of the system are non-linear and mimic corticospinal systems (such as a sigmoidal function that represents the cortico-muscular pathway). This is supported by our earlier study that explored how NIBS affects motor response times (4). In addition, although we assume the separability of the data is possible, we do not completely assume that the dynamic responses are entirely deconvolute. Instead, we posit that the separability of the system will be measurable and specifically the dynamic response and proportional outcomes measured though TMS or NMES are representative of the changes to those neural networks. We also do not explicitly state this as a final solution to the problem, but instead the first representative step to create tangible methods that can be used to fully investigate the corticospinal system.

## Data availability statement

The original contributions presented in the study are included in the article/Supplementary material, further inquiries can be directed to the corresponding author.

## Author contributions

FS was responsible for writing the manuscript and building the mechanical portion of the HMI. JM was responsible for writing portions of the manuscript and conducting the experiments. GS and SS wrote portions of the manuscript and helped to develop the clinical protocol. YA and MM developed the GUI and portions of the HMI. AT assisted with developing the HMI and reviewed the manuscript. All authors reviewed, edited, and agreed on the presentation of the manuscript.

## Funding

This project was funded by the National Science Foundation—Disability and Rehabilitation Engineering (award # 2130651).

## Conflict of interest

The authors declare that the research was conducted in the absence of any commercial or financial relationships that could be construed as a potential conflict of interest.

## Publisher’s note

All claims expressed in this article are solely those of the authors and do not necessarily represent those of their affiliated organizations, or those of the publisher, the editors and the reviewers. Any product that may be evaluated in this article, or claim that may be made by its manufacturer, is not guaranteed or endorsed by the publisher.

## Figures and Tables

**Figure A1:**
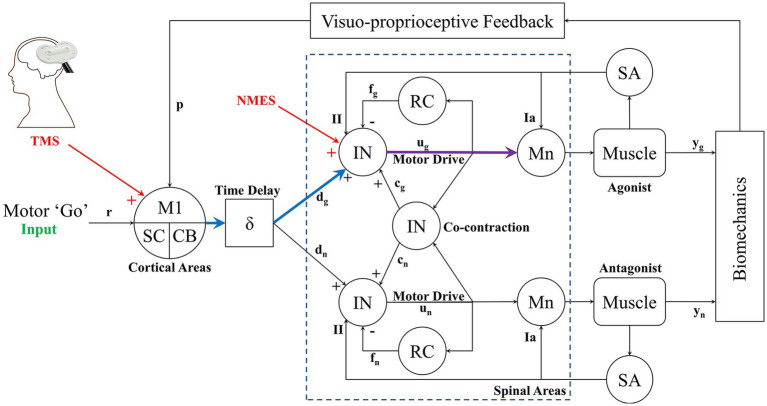
Computational framework of the complete corticospinal model. The model includes primary motor cortex (M1), brain stem areas (SC), cerebellum (CB) and spinal topologies provided by interneuron (IN), Renshaw cells (RC), motoneurons (Mn) and muscles. Afferent proprioceptive sensory information provided by Type-Ia and II are shown as well. Feedback is also given through visual perception of the movement. The cortical level (TMS) and spinal level (NMES) neurostimulation is shown that stimulate the cortical (blue) and spinal (purple) pathways that trigger muscle contraction.

## Appendix

Based on our team’s earlier research using tDCS, TMS, and NMES we have developed a computational representation (Figure A1) of the corticospinal system that served as rationale for our method described above. We use this model as our interpretation, which includes cortical areas such as the primary motor cortex (M1), cerebellum (CB), superior colliculus, and other brainstem areas (SC). At the spinal level interneurons (IN) connect to other IN involved in sensory-integration and coordination as well as motoneurons (Mn) that trigger muscular activity, Renshaw cells that provide recurrent feedback of Mn activity, and proprioceptors (SA) that provide Ia (muscle stretch velocity) and II afferent (stretch length), along with muscle tension (inherent to muscle block) in the model. The resultant muscle contractions based on these information drive movement biomechanics which is captured via proprioceptive feedback and visual information.

The cortico-muscular pathway triggered through TMS is shown in blue/purple, while the spinal-muscular pathway is shown in purple. Note that we show stimulation of the agonist muscle in this case, but mapping TMS and/or NMES to the antagonist is also possible. So, although our Equations 1–9 suggest a feedforward mechanism in motor control, the feedback responses will also affect motor response, however the feedback mechanism (such as in motor learning or cortical reprocessing) occurs at a much longer latency than the TMS or NMES stimulation. Thus the method we present provides an approach to probe instantaneous cortico-muscular or spino-muscular activity. But also provides a template for neuroplastic changes due to the feedback loops. For example, using these measurements, an individual’s neuromuscular responses can be tracked over time to see how cortical or spinal level motor control processing is modified. This then gives the method additional application in long-term testing of an individual’s neuromuscular adaptation or deterioration. A complete description of this system and its components can be found in our earlier studies (4, 68, 69).
