# Supplementary material for: A blended neurostimulation protocol to delineate cortico-muscular and spino-muscular dynamics following neuroplastic adaptation
Source: Front Neurol. 2023 Jun 15;14:1114860. doi: 10.3389/fneur.2023.1114860 (PMC10311503; doi:10.3389/fneur.2023.1114860)
Supplement: Supplementary file 1 [file Table_1.DOCX]

Supplemental Materials

HMI Code

% ModScript_loadcellDirect.m

%%%%%%%%%% This Script is for initial testing: There are 20 tests. the first 10 being random targets for flexion and the second 10 being random targets for extension

%%%%%%%%%%%% For each individual test, an excel file is saved with both time and cursor data, and a file is created for the entire stream of data as well

%sca; close all; clear; %Clear everything out

clearvars -except HMI; %deletes all variables except X in workspace

if exist('HMI', 'var') == 0

a= arduino('com3','Mega2560','libraries','advancedHX711/advanced_HX711');

HMI = addon (a,'advancedHX711/advanced_HX711','Pins',{'D3','D2'},'Interrupt',true, 'Gain',128);

end

cal = calibration(100,.1);

scale(cal,HMI);

tare(cal,HMI);

format short g;

%Values that can be changed accordingly

testAmount = 60; %Total number of tests to be taken, always keep even

timebetweentests = 2; %Number of seconds between each test

numTest = 0;

subdata = Subject_data; %Call to function for taking subject data

timeStart = datestr(now,'HH:MM:SS.FFF');

tic;

if subdata.cal == 'Y' || subdata.cal == 'y'

[factUp, factDown,calTime, timerCal] = SubjectCalibration_V3(toc, HMI,cal);

starter = length(calTime);

if factDown == 0

factDown = factUp/2;

end

elseif subdata.cal == 'N' || subdata.cal == 'n'

calTime = [];

timerCal = 0;

if subdata.domhand == 'r'

factUp = 5; %Increasing this number makes it more difficult to reach the selected target, absolute maximum around 60. Value gives maximum lb reading from load cell

factDown = 10;

elseif subdata.domhand == 'l'

factUp = 10;

factDown = 5;

end

end

scaleFactor = 10;

%Just initializng some global variables

exit = 0;

hit = 0;

%Make an identifier for the escape key

KbName('UnifyKeyNames');

escape = KbName('escape'); %Variable escape is associated with the key named escape

ret = KbName('return');

j = 1;

setupData = zeros(10000, 2);

cursorData = zeros(100000,2);

%%%%%%%%fullSwing = zeros(10000, 2);

falseSwing = zeros(10000,2);

%passArray = zeros(60, 1);

%fullSwing(1,1:starter) = calTime(:,1)';

%fullSwing(2, 1:starter) = calTime(:,2)';

Fs = 14400;

seconds = 0.5;

t = linspace(0, seconds, Fs*seconds + 1); % Time Vector + 1 sample

t(end) = []; % remove extra sample

freq = 500;

w = 2*pi*freq;

s = sin(w*t);

sEnd = sin(2*pi*200*t);

hitCount = 0; %Raw number corresponding to time on target

PsychDefaultSetup(2); %Just normal setup code for PsychToolBox

Screen('Preference', 'SkipSyncTests', 1); %Tell toolbox to skip sync tests since computer does not meet all requirements

screens = Screen('Screens'); %Create a variable for all of the available screens

screenNumber = max(screens); %Connect to outermost screen

black = BlackIndex(screenNumber); %Create the color black for the corresponding screen

white = WhiteIndex(screenNumber); %Create the color white for the corresponding screen

[window, windowRect] = PsychImaging('OpenWindow', screenNumber, black); %Find the size of the first window to be opened

[screenXpixels, screenYpixels] = Screen('WindowSize', window);

ifi = Screen('GetFlipInterval', window); %Get the 'flip' interval of screen, this is just a time between screen refreshes

[xCenter, yCenter] = RectCenter(windowRect); %Get the x and y coordinates of the center of the screen

targRange = setdiff(1:screenYpixels, (yCenter-100):(yCenter+100)); %Just creates a range of values for the target to be randomized from later. Notice it excludes the center point where the rectangle will start each test

%colorRange = 1:255;

xBase = screenXpixels;yBase = 200; %Set the size in x and y pixels of the target

setupVar = 1;

setupTargY = yCenter + 200;

baseSetupTarg = [0 0 xBase yBase];

centeredStartTarg = CenterRectOnPointd(baseSetupTarg, xCenter, setupTargY);

targSetColor = [0 0 1];

targIn = 0;

passRow = 1;

targY = yCenter + 400; %Set the y point of the first target

baseTarg = [0 0 xBase yBase]; %Set dimensions of target

centeredTarg = CenterRectOnPointd(baseTarg, xCenter, targY); %Set centerpoint of target

targColor = [.1 .4 .85]; %Set color of target

baseRect = [0 0 screenXpixels 20]; %Set the size of the cursor, it extends the full length

centeredRect = CenterRectOnPointd(baseRect, xCenter, yCenter); %Center the rectangle at the middle of the screen

rectColor = [.75 0 .25]; %Set the RGB co lor of the rectangle

failLineR = [0 0 screenXpixels 10];

failLine = CenterRectOnPointd(failLineR, xCenter, yCenter + (targY-yCenter)/4);

failColor = [1 0 0];

randflex = 1;

randext = 1;

%Starting Screen

Screen('TextSize', window, 90); %Set the font size of the displayed text

Screen('TextFont', window, 'Times'); %Set the font of the displayed text

%This part is just a screen that displays between each test to show which

%test was completed and the time until the next test

for i = 3:-1:1

DrawFormattedText(window, sprintf('Test run in %d s', i), 'center', 'center', white);

Screen('Flip', window);

pause(1);

end

sound(s, Fs);

k = 1;

while setupVar < 4 && numTest == 0

if subdata.domhand == 'r'

yRectCenter = get_weight(cal,HMI)*7050* (screenYpixels / 2 / scaleFactor) + (yCenter); %Change scale factor as needed

else

yRectCenter = -get_weight(cal,HMI)*7050* (screenYpixels / 2 / scaleFactor) + (yCenter); %Change scale factor as needed

end

if yRectCenter < (setupTargY + yBase/2) && yRectCenter > (setupTargY - yBase/2)

hitCount = hitCount + 1;

if(hitCount > 15) %Change back to 15

targSetColor = black;

hitCount = 0;

setupVar = setupVar + 1;

if (setupVar ~= 4)

Screen('FillRect', window, targSetColor, centeredStartTarg);

Screen('Flip', window);

targSetColor = [0 0 1];

pause(0.5);

sound(s, Fs);

end

end

else

hitCount = 0;

end

setupData(k,1) = toc; %Do not change this one. Takes actual seconds as reading.

setupData(k,2) =-yRectCenter + yCenter;

%fullSwing(1, k - 2) = toc + timerCal;

%fullSwing(2, k - 2) = -yRectCenter+yCenter;

k = k + 1;

centeredRect = CenterRectOnPointd(baseRect, xCenter, yRectCenter);

Screen('FillRect', window, targSetColor, centeredStartTarg);

Screen('FillRect', window, rectColor, centeredRect);

Screen('Flip', window);

end

setupData = setupData(1:k,:);

fullCount = k - 1;

if calTime ~= true

tester(:,1) = [calTime(:,1);setupData(:, 1)];

tester(:,2) = [calTime(:,2);setupData(:, 2)];

else

tester(:,1) = [setupData(:,1)];

tester(:,2) = [setupData(:,2)];

end

%plot(tester);

clicker = length(tester);

%%%%%%%fullSwing(1:length(tester),1) = tester(:,1);

%%%%%%%%%%%%fullSwing(1:length(tester),2) = tester(:,2);

falseCount = length(tester);

falseSwing(1:length(tester),1) = tester(:,1);

falseSwing(1:length(tester),2) = tester(:,2);

for i = 3:-1:1

DrawFormattedText(window, sprintf('Test Begins in %d s', i), 'center', screenYpixels * 0.25, white);

DrawFormattedText(window, 'Press escape to cancel test', 'center', screenYpixels * 0.75, white);

Screen('Flip', window);

loopRead = get_weight(cal,HMI)*7050*(screenYpixels/2/scaleFactor);

if (subdata.domhand=='l')

loopRead = -loopRead;

end

falseSwing(falseCount,1) = toc;

falseSwing(falseCount,2) = loopRead;

falseCount = falseCount + 1;

pause(1);

end

%Draw previously created target and rectangle

Screen('FillRect', window, targColor, centeredTarg);

Screen('FillRect', window, rectColor, centeredRect);

Screen('FillRect', window, failColor, failLine);

vbl = Screen('Flip', window);

waitframes = 1; %Set the number of frames to wait between refreshing screens

yRectCenter = yCenter; %Set the y coordinate of the cursor to be the center of the screen

%Take in readings until either escape is pressed or all of the tests are completed

sound(s,Fs);

while exit == 0 && numTest < testAmount %Checks global variables that indicate if either condition has been met

[keyIsDown, secs, keyCode, deltaSecs] = KbCheck; %Check if escape is pressed

%vy = readVoltage(a, 'A0');

if hit == 1 %Checks whether or not the target has been 'hit'

%Not important, just shrinks target once hit

passArray(passRow, 1)=1;

passRow=passRow+1;

for i = xBase:-100:0

baseTarg = [0 0 i yBase];

centeredTarg = CenterRectOnPointd(baseTarg, xCenter, targY);

if (numTest <= testAmount/3)

DrawFormattedText(window, 'Release & Relax', 'center', screenYpixels * 0.25, white);

elseif (numTest > testAmount/3) && (numTest <=testAmount*2/3)

DrawFormattedText(window, 'Release & Relax', 'center', screenYpixels * 0.75, white);

else

if targY>yCenter

DrawFormattedText(window, 'Release & Relax', 'center', screenYpixels * 0.25, white);

else

DrawFormattedText(window, 'Release & Relax', 'center', screenYpixels * 0.75, white);

end

end

Screen('FillRect', window, rectColor , centeredTarg);

Screen('Flip', window);

end

loopRead = get_weight(cal,HMI)*7050*(screenYpixels/2/scaleFactor);

if (subdata.domhand=='l')

loopRead = -loopRead;

end

falseSwing(falseCount,1) = toc;

falseSwing(falseCount,2) = loopRead;

falseCount = falseCount + 1;

%Displays screen between tests and the next test

if (numTest <= testAmount/3)

if (numTest < testAmount/3)

for i = timebetweentests:-1:1

Screen('TextSize', window, 90);

Screen('TextFont', window, 'Times');

count = sprintf('Next test in %d s', i);

DrawFormattedText(window, sprintf('Test %d complete', numTest), 'center', screenYpixels * 0.25, white);

DrawFormattedText(window, count, 'center', screenYpixels * 0.75, white);

Screen('Flip', window);

loopRead = get_weight(cal,HMI)*7050*(screenYpixels/2/scaleFactor);

if (subdata.domhand=='l')

loopRead = -loopRead;

end

falseSwing(falseCount,1) = toc;

falseSwing(falseCount,2) = loopRead;

falseCount = falseCount + 1;

pause(1);

end

targY = randi([yCenter+(screenYpixels/4) screenYpixels]);

elseif (numTest == testAmount/3)

for i = 10:-1:1

Screen('TextSize', window, 90);

Screen('TextFont', window, 'Times');

count = sprintf('Next test in %d s', i);

DrawFormattedText(window, 'Take a rest', 'center', screenYpixels*0.5, white )

DrawFormattedText(window, sprintf('Test %d complete', numTest), 'center', screenYpixels * 0.25, white);

DrawFormattedText(window, count, 'center', screenYpixels * 0.75, white);

Screen('Flip', window);

loopRead = get_weight(cal,HMI)*7050*(screenYpixels/2/scaleFactor);

if (subdata.domhand=='l')

loopRead = -loopRead;

end

falseSwing(falseCount,1) = toc;

falseSwing(falseCount,2) = loopRead;

falseCount = falseCount + 1;

pause(1);

end

targY = randi([0 yCenter-(screenYpixels/4)]);

end

elseif (numTest > testAmount/3) && (numTest <= testAmount * 2/3)

if (numTest > testAmount/3) && (numTest<testAmount *2/3)

for i = timebetweentests:-1:1

Screen('TextSize', window, 90);

Screen('TextFont', window, 'Times');

count = sprintf('Next test in %d s', i);

DrawFormattedText(window, sprintf('Test %d complete', numTest), 'center', screenYpixels * 0.25, white);

DrawFormattedText(window, count, 'center', screenYpixels * 0.75, white);

Screen('Flip', window);

loopRead = get_weight(cal,HMI)*7050*(screenYpixels/2/scaleFactor);

if (subdata.domhand=='l')

loopRead = -loopRead;

end

falseSwing(falseCount,1) = toc;

falseSwing(falseCount,2) = loopRead;

falseCount = falseCount + 1;

pause(1);

end

targY = randi([0 yCenter-(screenYpixels/4)]);

elseif (numTest == testAmount *2/3)

for i = 10:-1:1

Screen('TextSize', window, 90);

Screen('TextFont', window, 'Times');

count = sprintf('Next test in %d s', i);

DrawFormattedText(window, 'Take a rest', 'center', screenYpixels*0.5, white )

DrawFormattedText(window, sprintf('Test %d complete', numTest), 'center', screenYpixels * 0.25, white);

DrawFormattedText(window, count, 'center', screenYpixels * 0.75, white);

Screen('Flip', window);

loopRead = get_weight(cal,HMI)*7050*(screenYpixels/2/scaleFactor);

if (subdata.domhand=='l')

loopRead = -loopRead;

end

falseSwing(falseCount,1) = toc;

falseSwing(falseCount,2) = loopRead;

falseCount = falseCount + 1;

pause(1);

end

targY = randi([0 yCenter-(screenYpixels/4)]);

if(targY > yCenter)

randflex = randflex + 1;

elseif (targY < yCenter)

randext = randext + 1;

end

end

elseif (numTest > testAmount*2/3)

for i = timebetweentests:-1:1

Screen('TextSize', window, 90);

Screen('TextFont', window, 'Times');

count = sprintf('Next test in %d s', i);

DrawFormattedText(window, sprintf('Test %d complete', numTest), 'center', screenYpixels * 0.25, white);

DrawFormattedText(window, count, 'center', screenYpixels * 0.75, white);

Screen('Flip', window);

loopRead = get_weight(cal,HMI)*7050*(screenYpixels/2/scaleFactor);

if (subdata.domhand=='l')

loopRead = -loopRead;

end

falseSwing(falseCount,1) = toc;

falseSwing(falseCount,2) = loopRead;

falseCount = falseCount + 1;

pause(1);

end

if mod(numTest,2) == 0

targY = randi([0 yCenter-(screenYpixels/4)]);

else

targY = randi([yCenter+(screenYpixels/4) screenYpixels]);

end

if(targY > yCenter)

randflex = randflex + 1;

elseif (targY < yCenter)

randext = randext + 1;

end

end

sound(s, Fs);

sparf = fullCount;

fullCount = fullCount + j - 1;

targColor = [0 0 255]; %RGB color of target

baseTarg = [0 0 xBase yBase]; %This just gives the size of the target. This doesn't change at all

pause(0.5); %Just pauses the program between tests

hit = 0; %Puts the hit global variable back at zero for the next hit

j = 1;

cursorData = zeros(1000,2); %Empty out array for next data set

yRectCenter = get_weight(cal,HMI)*7050 * (screenYpixels / 2 / scaleFactor) + (yCenter); %Change scale factor as needed

end

%Takes in a reading from the serial connection to arduino, scales it to +- direction of screen pixels, and then just moves it so that the center of the screen is zero

if (subdata.domhand == 'r')

if(numTest<=testAmount/3)

scaleFactor = factDown;

elseif (numTest> testAmount/3) && (numTest <= testAmount*2/3)

scaleFactor = factUp;

elseif (numTest>testAmount*2/3)

if(targY > yCenter)

scaleFactor = factDown;

else

scaleFactor = factUp;

end

end

yRectCenter = get_weight(cal,HMI)*7050 * (screenYpixels / 2 / scaleFactor) + (yCenter); %Change scale factor as needed

elseif (subdata.domhand == 'l')

if(numTest<=testAmount/3)

scaleFactor = factUp;

elseif(numTest>testAmount/3) && (numTest <= testAmount*2/3)

scaleFactor = factDown;

elseif (numTest>testAmount*2/3)

if(targY>yCenter)

scaleFactor = factUp;

else

scaleFactor = factDown;

end

end

yRectCenter = -get_weight(cal,HMI)*7050 * (screenYpixels / 2 / scaleFactor) + (yCenter); %Change scale factor as needed

end

j = j + 1;

%Two ways to save data:

timerTest = toc;

cursorData(j, 1) = timerCal + timerTest; %Do not change this one. Takes actual seconds as reading.

cursorData(j, 2) =-yRectCenter + yCenter; %First, save the position of the cursor as seen on the screen

%This chunk just prevents the cursor from leaving the screen

if yRectCenter > screenYpixels - 20

yRectCenter = screenYpixels - 20;

elseif yRectCenter < 20

yRectCenter = 20;

end

%Creates a rectangle and a target for previously specified dimensions and centers them at x and y coordinates

%Target bounds do not change, but the centerpoint of the cursor is what gets altered for each loop reading

if targY > yCenter

failY = yCenter + 0.25*(targY-yCenter);

else

failY = yCenter -50 +0.25*(targY-yCenter);

end

if targIn == 1

if(targY > yCenter)

if (yRectCenter < failY)

Screen('FillRect', window, black, [0 0 screenXpixels screenYpixels]);

DrawFormattedText(window, 'Relax', 'center', 'center', white);

Screen('Flip', window);

targIn = 0;

pause(1);

passArray(passRow, 1) = 0;

passRow = passRow + 1;

end

elseif(targY<yCenter)

if(yRectCenter > failY)

Screen('FillRect', window, black, [0 0 screenXpixels screenYpixels]);

DrawFormattedText(window, 'Relax', 'center', 'center', white);

Screen('Flip', window);

targIn = 0;

pause(1);

passArray(passRow, 1) = 0;

passRow = passRow + 1;

end

else

targIn = 0;

end

Screen('FillRect', window, black, [0 0 screenXpixels screenYpixels]);

end

centeredRect = CenterRectOnPointd(baseRect, xCenter, yRectCenter);

centeredTarg = CenterRectOnPointd(baseTarg, xCenter, targY);

failLine = CenterRectOnPointd(failLine, xCenter, failY);

Screen('FillRect', window, targColor, centeredTarg);

Screen('FillRect', window, rectColor, centeredRect);

Screen('FillRect', window, failColor, failLine);

if targY > yCenter

if yRectCenter > failY

targIn = 1;

end

elseif targY < yCenter

if yRectCenter < failY

targIn = 1;

end

end

%If the cursor is within range of the target, start a timer and if that timer is reached, then consider the target as 'hit' and move onto the next test

if yRectCenter < (targY + yBase/2) && yRectCenter > (targY - yBase/2)

targIn = 1;

hitCount = hitCount + 1;

if(hitCount > 15) %Change back to 15

targIn = 0;

numTest = numTest + 1;

hit = 1;

%col1 = randi(255);col2 = randi(255);col3 = randi(255);

hitCount = 0;

testData = cursorData(1:j, :);

end

else

hitCount = 0;

end

if keyCode(escape) == 1 %If escape is pressed, just break the while loop

exit = 1;

elseif keyCode(ret) == 1

numTest = numTest + 1;

end

%%%fullSwing(clicker, 1) = toc;

%%%%%fullSwing(clicker, 2) = yCenter-yRectCenter;

clicker = clicker + 1;

falseSwing(falseCount,1) = toc;

falseSwing(falseCount,2) = yCenter-yRectCenter;

falseCount = falseCount + 1;

vbl = Screen('Flip', window, vbl + (waitframes - 0.5) * ifi); %Draw the screen and refresh it as needed

end

%The loop either broke because all tests were complete or because the escape key was pressed. Just indicate that to the user

sound(sEnd, Fs);

pause(0.5);

timeStop= datestr(now,'HH:MM:SS.FFF');

falseSwing(falseCount,1) = toc;

loopRead = get_weight(cal,HMI)*7050*(screenYpixels/2/scaleFactor);

if (subdata.domhand=='l')

loopRead = -loopRead;

end

falseSwing(falseCount,1) = toc;

falseSwing(falseCount,2) = loopRead;

falseCount = falseCount + 1;

%%%%fullSwing = fullSwing(1:clicker-1,:);

falseSwing = falseSwing(1:falseCount-1,:);

if numTest >= testAmount

passArray(passRow, 1) = 1;

DrawFormattedText(window, 'All Tests Complete', 'center', screenYpixels * 0.33, [.50 .40 .6]);

DrawFormattedText(window, 'End of session', 'center', screenYpixels * 0.67, [.50 .40 .6]);

else

DrawFormattedText(window, 'Test Canceled', 'center', screenYpixels * 0.25, [1 0 0]);

DrawFormattedText(window, 'closing program', 'center', screenYpixels * 0.75, [1 0 0]);

end

sparf = fullCount;

fullCount = fullCount + j - 1;

fullCursor = sprintf('Sub%s_trial%s_FullStream.xlsx', subdata.nr, subdata.trial);

sequence = sprintf('Sub%s_%s_Sequence', subdata.nr, subdata.trial);

dataTime = sprintf('Sub%s_%s_Time', subdata.nr,subdata.trial);

writematrix([timeStart; timeStop],dataTime);

% fullSwing = fullSwing(:, 1:fullCount);

writematrix(falseSwing, fullCursor);

writematrix(passArray', sequence);

Screen('Flip', window); %Draw a screen based on how the loop was broken

pause(2); %Show the screen for two seconds

sca; %Clear all screens %Clear all screens
